# Effects of pre-membrane acidification on extracorporeal CO_2_ removal compared with conventional ECCO_2_R in mechanically ventilated animal models: a preclinical systematic review protocol

**DOI:** 10.62675/2965-2774.20260105

**Published:** 2026-09-28

**Authors:** Vagner Pires de Campos, Anne Caroline de Almeida Sousa, Yuri de Albuquerque Pessoa dos Santos, Maristela Carvalho, Marcelo Amato Britto Passos, Eduardo Leite Vieira Costa

**Affiliations:** 1 Universidade de São Paulo Faculdade de Medicina São Paulo SP Brazil Faculdade de Medicina, Universidade de São Paulo - São Paulo (SP), Brazil; 2 Hospital Sírio-Libanês São Paulo SP Brazil Intensive Care Unit, Hospital Sírio-Libanês - São Paulo (SP), Brazil; 3 Universidade de São Paulo Hospital das Clínicas Faculdade de Medicina São Paulo SP Brazil Medical Intensive Care Unit, Hospital das Clínicas, Faculdade de Medicina, Universidade de São Paulo - São Paulo (SP), Brazil; 4 Instituto do Coração Universidade de São Paulo Hospital das Clínicas São Paulo SP Brazil Pulmonary Division, Instituto do Coração, Hospital das Clínicas, Faculdade de Medicina, Universidade de São Paulo - São Paulo (SP), Brazil

**Keywords:** Extracorporeal life support, Respiratory insufficiency, Animals

## Abstract

**Objective:**

To systematically evaluate the effect of pre-membrane blood acidification, compared with conventional extracorporeal carbon dioxide removal without acidification, on extracorporeal - carbon dioxide elimination in large animal models undergoing mechanical ventilation.

**Methods:**

This protocol is prospectively registered in PROSPERO and will be conducted in accordance with PRISMA-P guidelines. We will include preclinical experimental studies in large animals (e.g., swine, sheep, goats, cattle, or dogs) evaluating pre-membrane acidification associated with extracorporeal carbon dioxide removal. We will conduct electronic searches in PubMed^®^, Web of Science, Scopus, Embase, and Google Scholar from database inception to December 2025, without language restrictions. Two independent reviewers will conduct study selection, data extraction, and risk-of-bias assessment using the SYRCLE tool. The primary outcome will be extracorporeal carbon dioxide elimination. When appropriate, we will conduct meta-analyses using random-effects models. We will standardize carbon dioxide elimination values by body weight (mL/kg/minute) to improve comparability across species.

**Results:**

This review will provide a structured synthesis of preclinical evidence regarding the magnitude and consistency of the effect of pre-membrane acidification on carbon dioxide elimination, ventilatory parameters, and adverse events.

**Conclusion:**

By systematically evaluating available experimental data, this review aims to clarify the potential role of pre-membrane acidification as an adjunctive strategy to extracorporeal carbon dioxide removal and to identify knowledge gaps relevant for future translational and clinical research.

## INTRODUCTION

Extracorporeal carbon dioxide (CO_2_) removal (ECCO_2_R) is a strategy developed to assist in the management of hypercapnic respiratory failure, promoting lung protection and reducing the deleterious effects of invasive mechanical ventilation, particularly in cases of acute respiratory distress syndrome (ARDS) and chronic obstructive pulmonary disease (COPD). ^( 1 , 2 )^

However, although the literature demonstrates reductions in ventilatory parameters with ECCO^2^R support, outcomes such as ventilator-free days and mortality remain uncertain. ^( 3 , 4 )^ Several methodological factors, including patient selection, optimal timing of therapy initiation, anticoagulation strategies, and the occurrence of adverse events, limit the consistency and generalizability of results. ^( 4 )^

Accordingly, further investigations have sought to identify factors associated with CO_2_ removal during therapy and strategies to optimize them. In an experimental study with preliminary findings, Santos et al. identified sweep gas flow as the main variable associated with CO_2_ transfer during therapy when combined with continuous renal replacement therapy using high bicarbonate concentrations. ^( 3 )^ In addition, hemoglobin levels, oxygen saturation, body temperature, and pre-membrane partial pressure of CO_2_ were also identified as modulatory variables influencing CO_2_ removal. ^( 5 )^

In this context, pre-membrane acidification techniques have been proposed to enhance CO_2_ removal at low blood flow rates. Acidification promotes the conversion of circulating CO_2_ – primarily present in the form of bicarbonate – into dissolved CO_2_ and water in the presence of hydrogen ions. ^( 6 )^ Different acidification methods have been described, including infusion of lactic or citric acid, cation exchange resins, and electrodialysis. ^( 7 - 9 )^ The findings of these studies reinforce the potential of pre-membrane acidification as an adjunctive strategy to conventional ECCO_2_R, demonstrating increased carbon dioxide elimination (VCO_2_) in mechanically ventilated animal models.

Despite these promising findings, recent narrative reviews emphasize that different blood acidification methods remain largely limited to preclinical settings and proof-of-concept studies. ^( 2 , 10 )^ Relevant uncertainties persist regarding the effectiveness of this strategy in increasing ${\dot{\text{V}}\text{CO}}_{2}$ , the incidence of adverse events, and the reproducibility of different methods across distinct experimental contexts. Furthermore, the marked heterogeneity of acidification techniques – including infusion of organic acids (such as lactic or citric acid), use of cation exchange resins, and reduction of bicarbonate concentration – creates an additional barrier to comparative interpretation of results and to translating experimental findings into clinical practice. To date, no systematic review has critically synthesized the preclinical evidence regarding pre-membrane acidification associated with ECCO^2^R.

Given the lack of a systematic synthesis of available preclinical evidence, this study proposes a systematic review protocol to evaluate the effect of pre-membrane blood acidification - regardless of the technique employed – compared with conventional ECCO_2_R without acidification, on extracorporeal CO_2_ elimination through the membrane ( ${\dot{\text{V}}\text{CO}}_{2}$ ) in animal models. The specific objectives of this review are: to compare the effect of pre-membrane acidification with conventional ECCO_2_R on CO_2_ removal; to evaluate the impact of acidification techniques on respiratory and hemodynamic variables; to describe and characterize the different pre-membrane acidification methodologies reported in the literature, outlining their respective technical limitations; to analyze the incidence of adverse events and complications associated with acidification techniques; to identify knowledge gaps and critical barriers to the translation of preclinical findings into clinical practice.

## METHODS

This protocol is prospectively registered in the International Prospective Register of Systematic Reviews (PROSPERO) database (CRD420261334780) and follows the Cochrane Handbook for Systematic Reviews of Interventions. A search was conducted in PROSPERO (using the "Search PROSPERO" field) on December 14, 2025, using the following terms: (ECCO2R OR "extracorporeal CO2 removal" OR "extracorporeal carbon dioxide removal") AND (acidification OR "blood acidification" OR "pre-membrane acidification" OR premembrane), in addition to separate searches using ECCO2R and acidification. No prospective registrations clearly corresponding to the topic of this review (pre-membrane acidification associated with ECCO_2_R in animal models, with ${\dot{\text{V}}\text{CO}}_{2}$ as outcome) were identified. In addition, this protocol follows the Preferred Reporting Items for Systematic Reviews and Meta-Analyses Protocols (PRISMA-P). The review process started in December 2025 and is expected to be completed by December 2026.

The PICOS question of this systematic review is as follows: in experimental models involving large animals (e.g., swine, sheep, goats, or dogs) undergoing mechanical ventilation and extracorporeal CO_2_ removal, does pre-membrane blood acidification, compared with conventional ECCO_2_R without acidification, increase extracorporeal CO_2_ elimination through the membrane ( ${\dot{\text{V}}\text{CO}}_{2}$ )?

This review will restrict inclusion to large animal models for translational relevance and external validity, as these models more closely approximate clinical ECCO_2_R conditions in humans.

### Context and concept

#### Patient and public involvement

No patients were involved.

#### Type of studies

We will include preclinical experimental studies conducted in large animal models with controlled designs, including proof-of-concept studies, whether randomized or non-randomized, that evaluate the application of pre-membrane blood acidification in association with ECCO_2_R. We will consider both parallel and sequential experimental designs (e.g., before-after or cross-over), provided they include a comparator group or condition corresponding to conventional ECCO_2_R without acidification and allow extraction of quantitative data on extracorporeal CO_2_ elimination through the membrane ( ${\dot{\text{V}}\text{CO}}_{2}$ ).

We will exclude case series, case reports, exclusively *in vitro* or *ex vivo* studies, purely methodological or bench studies, reviews, editorials, letters, comments, and protocols, as well as experimental studies that do not present an appropriate comparator group or condition. We will include both published and unpublished studies. Unpublished evidence will encompass conference abstracts, theses, dissertations, preprints, and other forms of grey literature, provided that sufficient methodological and outcome data are available. No restrictions will be applied regarding language or publication date.

#### Type of participants

We will include experimental models involving large animals (e.g., adult swine, sheep, goats, cattle, or dogs) undergoing invasive mechanical ventilation and ECCO_2_R. Studies conducted in humans, as well as those involving neonatal or pregnant specimens, will be excluded. Studies using extracorporeal membrane oxygenation (ECMO) will be included only if they include a clearly defined ECCO_2_R arm with pre-membrane acidification and analyze it separately.

#### Type of interventions

We will include experimental studies in large animals undergoing invasive mechanical ventilation and ECCO_2_R with pre-membrane blood acidification, regardless of the method used (e.g., acid infusion, cation exchange resins, or reduction of bicarbonate concentration). All ventilatory modes will be considered.

The study must explicitly describe and characterize ECCO_2_R use. Studies must report quantitative extracorporeal carbon dioxide elimination through the membrane ( ${\dot{\text{V}}\text{CO}}_{2}$ ) or provide sufficient data to calculate ${\dot{\text{V}}\text{CO}}_{2}$ according to the reported method (e.g., sweep gas flow and CO_2_ fraction in exhaust gas; or pre- and post-membrane blood gas parameters, blood flow, pH, and PaCO_2_). Studies using ECMO will be included only when a clearly defined experimental arm characterized as ECCO_2_R with pre-membrane acidification is available.

#### Type of outcome measures

The primary outcome will be ${\dot{\text{V}}\text{CO}}_{2}$ eliminated through the extracorporeal membrane (mL/minute). Secondary outcomes will include adverse events, driving pressure (cmH_2_O), tidal volume (mL/kg predicted body weight), systemic arterial pH, extracorporeal circuit pH, systemic arterial PaCO_2_ (mmHg), and extracorporeal circuit PaCO_2_ (mmHg).

### Search strategy

We will perform a comprehensive search with no restrictions regarding language, country of origin, or publication status. When needed, we will contact corresponding authors for clarification or additional data if study reports are unclear, and we will arrange translations when required. We will conduct searches from database inception to December 2025 and update them before final manuscript submission.

We will conduct electronic searches in PubMed^®^, Web of Science, Scopus, Embase, and Google Scholar using an adapted search strategy ( Table 1S - Supplementary Material ). To maximize retrieval of relevant studies, we will use supplementary search strategies, including snowballing (manual search of reference lists of included studies). We will use Artificial Intelligence-assisted citation mapping tools (e.g., Connect Papers and Litmaps) as complementary strategies for forward citation tracking.

The search strategy was systematically and comprehensively constructed by combining controlled vocabulary terms and free-text terms. Initially, descriptors were identified in the Medical Subject Headings (MeSH) and *Descritores em Ciências da Saúde* (DeCS) vocabularies and supplemented with additional terms extracted from relevant literature, including synonyms, spelling variations, and technical terminology related to the review topic. A librarian specialized in systematic search strategies reviewed the preliminary strategy, and it was refined after consultation with a subject-matter expert to ensure adequate sensitivity, terminological precision, and comprehensive coverage of the relevant literature.

### Study selection

After completion of the search, all identified citations will be compiled and imported into reference management software, such as EndNote. During this process, duplicate records will be removed. The resulting file will then be exported to Rayyan, where two independent reviewers will conduct the study selection process based on predefined eligibility criteria.

In Rayyan, reviewers will first conduct a pilot test independently and in a blinded manner using five references (titles and abstracts). Following this preliminary phase, study selection will proceed in two stages: screening of titles and abstracts, followed by full-text assessment, both guided by the established inclusion criteria.

In cases of disagreement regarding study inclusion or exclusion, a third reviewer will be consulted, and conflicts will be resolved through consensus meetings. If translation of abstracts or full texts into another language is required for eligibility assessment, we will use an online document translation tool.

We will describe the study selection process in sufficient detail to complete the PRISMA flow diagram. ^( 11 )^ Excluded studies, along with the reasons for exclusion, will be summarized in a table entitled "Characteristics of Excluded Studies."

### Data extraction

We will develop an initial data extraction template in Microsoft Excel. The team will refine and adjust this preliminary template as needed throughout data extraction for each included study, and document any modifications to the extraction form. Two reviewers (R1 and R2) will extract data independently and in a blinded manner using a data extraction tool developed by the authors. The extraction process will include collecting information aligned with the PICOS framework. The authors will resolve any discrepancies between reviewers with the involvement of a third reviewer (R3). When deemed necessary, study authors will be contacted to obtain missing data or additional information essential for the review. Studies derived from the same research project (e.g., a full article and a conference abstract) will be grouped, and only the full article will be considered for data extraction.

The following data will be extracted from each included study: general study characteristics (first author, year of publication, country, and funding source); animal model details (species, sex, weight, and experimental condition, including the presence and type of induced lung injury); ECCO_2_R technical parameters (blood flow, sweep gas flow, membrane surface area, and device characteristics); acidification strategy (type of acid or technique used, concentration or dose, site of application within the extracorporeal circuit); comparator characteristics; outcome measures with respective units (including ${\dot{\text{V}}\text{CO}}_{2}$ in mL/minute and secondary physiological parameters); time points of outcome assessment; and all numerical data required for effect size calculation (means, standard deviations [SD], sample size per group, or data enabling transformation).

### Risk of bias

Two reviewers will independently assess the risk of bias in the included studies. Because this systematic review will include only preclinical experimental studies in animal models, we will evaluate risk of bias using SYRCLE's Risk of Bias tool, which was specifically developed for animal studies. ^( 12 )^ This tool assesses the following domains: selection bias, performance bias, detection bias, attrition bias, reporting bias, and other sources of bias. ^( 13 )^

Prior to applying the tool, reviewers will discuss the instrument domains in detail and establish, by consensus, the criteria and parameters for their assessment. Subsequently, reviewers will conduct a calibration process until they achieve an optimal level of agreement (Kappa index ≥ 0.8). Both reviewers will then assess risk of bias independently, and a third evaluator will resolve any disagreements.

We will assess the reporting quality of included studies descriptively based on selected items from the ARRIVE guidelines for animal research. ^( 14 )^ This assessment will be complementary and will not be used as an exclusion criterion; rather, it will serve to contextualize the methodological transparency of the literature and assist in interpreting the risk of bias and strength of evidence. We will evaluate the potential impact of publication bias and small-study effects by visually inspecting funnel plots, provided a sufficient number of studies are available for each outcome (≥ 10 studies). We will interpret any observed asymmetry cautiously, considering the particular characteristics of preclinical studies, including methodological heterogeneity and differences in experimental models.

### Statistical plan

We will conduct a narrative synthesis, combining included studies for each comparison at the outcome level. If the included studies are sufficiently homogeneous with respect to PICOS, we will pool effect estimates with corresponding 95% confidence intervals (95%CI). We will perform quantitative synthesis only if at least two independent studies report comparable data for the same outcome. Otherwise, we will provide a structured narrative synthesis following the Synthesis Without Meta-analysis (SWiM) guidelines. We will present data in forest plots comparing conventional ECCO_2_R with acidification ECCO_2_R.

The primary comparison of interest will be acidification *versus* no acidification. While we assume a shared physiological mechanism via pH reduction, we acknowledge that different techniques may not be equivalent. Therefore, we will perform mandatory subgroup analyses by acidification method (e.g., acid infusion vs. cation exchange resins) to identify method-specific effects. Additional subgroup analyses will explore the impact of animal species and ECCO_2_R configurations.

To ensure biological comparability across different animal species and body sizes, VCO_2_ (values will be normalized by body weight (mL/kg/minute). When we report absolute values (mL/minute), we will convert them by dividing the mean VCO_2_ by the mean body weight of the experimental group. We will adjust the SD proportionally (SD {adj} = SD / mean body weight) to preserve the variance structure. If weight data are unavailable, we will analyze absolute values separately in sensitivity analyses.

We will pool individual study estimates using a random-effects meta-analysis model with the Hartung-Knapp-Sidik-Jonkman (HKSJ) estimator. This approach is pre-specified as it provides more conservative and robust confidence intervals for preclinical data with expected high heterogeneity.

Statistical heterogeneity will be assessed using I^2^ and tau^2^ statistic, which expresses the percentage of total variation due to real differences between studies rather than random error. Following the Cochrane Handbook guidelines, I^2^ values will be interpreted using the following thresholds as an approximate guide: 25% (low), 50% (moderate), and 75% (high) heterogeneity. Additionally, we will calculate the 95% prediction interval to estimate the expected effect in future experimental settings.

When heterogeneity is significant, we will perform subgroup analyses by acidification dose, acid type, and site of acidification (e.g., acidified dialysate *versus* direct blood acidification). To further explore sources of heterogeneity, we will conduct sensitivity analyses using the "leave-one-out" method, systematically removing each study to assess its individual impact on the overall effect estimate.

To address potential biases arising from study design, additional sensitivity analyses will compare randomized or cross-over designs against before-after (non-randomized) studies. Meta-regression will be conducted as an exploratory tool to evaluate the influence of acidification dose and other study-level variables (e.g., animal species and extracorporeal blood flow) on the primary outcome, provided at least ten studies are available.

When numerical data are missing, we will extract values from figures using WebPlotDigitizer or calculate them from available metrics (e.g., standard errors). We will contact authors for missing data, and sensitivity analyses will assess the impact of these estimates.

## DISCUSSION

This systematic review aims to synthesize the available preclinical evidence on the impact of pre-membrane acidification on extracorporeal CO_2_ removal ( ${\dot{\text{V}}\text{CO}}_{2}$ ) and associated ventilatory parameters. Experimental studies have suggested that acidification strategies may enhance CO_2_ elimination across the extracorporeal membrane and potentially allow further reductions in tidal volume and minute ventilation. ^( 7 , 8 )^

In the clinical setting, protective ventilation strategies have demonstrated that increased driving pressure and respiratory rate are associated with higher mortality in patients with ARDS. ^( 15 )^ Permissive hypercapnia has therefore been widely adopted to facilitate lung-protective ventilation. However, in severe forms of lung injury with markedly reduced compliance, the therapeutic window becomes limited, as effective CO_2_ clearance may not be achievable even with low tidal volumes. ^( 16 )^ Progressive hypercapnia may lead to clinically significant acidemia, potentially compromising the feasibility of ultra-protective ventilation strategies. ^( 17 , 18 )^ In this context, extracorporeal CO_2_ removal has emerged as a supportive alternative to facilitate lung protection. ^( 18 )^

Pre-membrane blood acidification represents a physiologically grounded strategy aimed at enhancing CO_2_ transfer by shifting the bicarbonate equilibrium toward dissolved CO_2_. Although proof-of-concept studies in large animal models suggest potential benefits, the available evidence remains heterogeneous and largely confined to preclinical settings.

This synthesis has two translational implications. First, by establishing a standardized effect size in mL/kg/minute, our findings will provide a benchmark for clinicians and researchers to assess whether the magnitude of carbon dioxide removal achieved in animal models is sufficient to support ultra-protective ventilation in humans. Second, this review will systematically map adverse events, such as hemolysis or hemodynamic instability, which remain critical barriers to clinical implementation. Furthermore, pre-membrane acidification is presented as a method to improve carbon dioxide removal efficiency and contribute to the adherence to lung-protective ventilation strategies.

This review will therefore quantify the effect of pre-membrane acidification on ${\dot{\text{V}}\text{CO}}_{2}$ and related physiological parameters, describe methodological variability across experimental models, and identify knowledge gaps that may inform future translational and clinical investigations.

Interpreting our findings must account for the substantial heterogeneity inherent in preclinical research, particularly in experimental protocols, acidification techniques, and the broad variability in animal species and body mass. To address these challenges, we implemented rigorous weight-based normalization of VCO_2_ (mL/kg/minute), which serves as a critical biological bridge to harmonize data across animal models. Furthermore, while grouping diverse acidification methods presents a potential limitation, our mandatory subgroup analyses and the Hartung-Knapp-Sidik-Jonkman estimator ensure a conservative, method-specific evaluation of efficacy. Despite the inherent constraints of translating these preclinical data to clinical practice, applying such rigorous methodological standards allows this review to clarify the magnitude and consistency of the reported effects. This approach provides a structured, evidence-based framework essential for informing the design and safety parameters of future translational research in extracorporeal CO_2_ removal.

## Data Availability

Not applicable. This article is a study protocol.

## References

[1] Lassola S, Battaglini D, De Rosa S (2025). Advancing extracorporeal carbon dioxide removal technology: bridging basic science and clinical practice. Med Gas Res.

[2] Lamas T, Fernandes SM, Vasques F, Karagiannidis C, Camporota L, Barrett N (2024). Recent advances and future directions in extracorporeal carbon dioxide removal. J Clin Med.

[3] Santos YA, Costa EL, Mendes PV, Park M, Besen BA (2026). Why have extracorporeal carbon dioxide removal clinical trials failed?. Crit Care Sci.

[4] Slutsky A, Brodie D (2026). The (broken) promise of extracorporeal carbon dioxide removal — failure or future?. Am J Respir Crit Care Med.

[5] Santos YA, Cardoso LC, Mendes PV, Besen BA, Park M (2024). Factors associated with carbon dioxide transfer in an experimental model of severe acute kidney injury and hypoventilation during high bicarbonate continuous renal replacement therapy and oxygenation membrane support. Crit Care Sci.

[6] Zanella A, Mangili P, Giani M, Redaelli S, Scaravilli V, Castagna L (2014). Extracorporeal carbon dioxide removal through ventilation of acidified dialysate: an experimental study. J Heart Lung Transplant.

[7] Takahashi N, Nakada TA, Sakai T, Kato Y, Moriyama K, Nishida O (2020). A CO^2^removal system using extracorporeal lung and renal assist device with an acid and alkaline infusion. J Artif Organs.

[8] Zanella A, Mangili P, Redaelli S, Scaravilli V, Giani M, Ferlicca D (2014). Regional blood acidification enhances extracorporeal carbon dioxide removal: a 48-hour animal study. Anesthesiology.

[9] Scaravilli V, Kreyer S, Linden K, Belenkiy S, Pesenti A, Zanella A (2015). Enhanced extracorporeal CO2 removal by regional blood acidification: effect of infusion of three metabolizable acids. ASAIO J.

[10] Boyle AJ, Sklar MC, McNamee JJ, Brodie D, Slutsky AS, Brochard L, International ECMO Network (ECMONet) (2018). Extracorporeal carbon dioxide removal for lowering the risk of mechanical ventilation: research questions and clinical potential for the future. Lancet Respir Med.

[11] Page MJ, McKenzie JE, Bossuyt PM, Boutron I, Hoffmann TC, Mulrow CD (2021). The PRISMA 2020 statement: an updated guideline for reporting systematic reviews. Rev Esp Cardiol (Engl Ed).

[12] Beck NB, Becker RA, Boobis AR, Fergusson D, Fowle JR, Goodman J (2014). Instruments for assessing risk of bias and other methodological criteria of animal studies: omission of well-established methods. Environ Health Perspect.

[13] Hooijmans CR, Rovers MM, de Vries RB, Leenaars M, Ritskes-Hoitinga M, Langendam MW (2014). SYRCLE's risk of bias tool for animal studies. BMC Med Res Methodol.

[14] Percie du Sert N, Hurst V, Ahluwalia A, Alam S, Avey MT, Baker M (2020). The ARRIVE guidelines 2.0: updated guidelines for reporting animal research. PLoS Biol.

[15] Costa EL, Slutsky AS, Brochard LJ, Brower R, Serpa-Neto A, Cavalcanti AB (2021). Ventilatory variables and mechanical power in patients with acute respiratory distress syndrome. Am J Respir Crit Care Med.

[16] Pelosi P, Ball L, Barbas CS, Bellomo R, Burns KE, Einav S (2021). Personalized mechanical ventilation in acute respiratory distress syndrome. Crit Care.

[17] Laffey JG, O’Croinin D, McLoughlin P, Kavanagh BP (2004). Permissive hypercapnia—role in protective lung ventilatory strategies. Intensive Care Med.

[18] McNamee JJ, Gillies MA, Barrett NA, Perkins GD, Tunnicliffe W, Young D, REST Investigators (2021). Effect of lower tidal volume ventilation facilitated by extracorporeal carbon dioxide removal vs standard care ventilation on 90-day mortality in patients with acute hypoxemic respiratory failure: the REST randomized clinical trial. JAMA.

