# Supplementary material for: Effects of pre-membrane acidification on extracorporeal CO2 removal compared with conventional ECCO2R in mechanically ventilated animal models: a preclinical systematic review protocol
Source: Crit Care Sci. 2026 Sep 28;38:e20260105. doi: 10.62675/2965-2774.20260105 (PMC13690042; doi:10.62675/2965-2774.20260105)
Supplement: Supplementary file 1 [file 2965-2774-ccsci-38-e20260105-suppl01.pdf]

Critical Care Science
https://doi.org/10.62675/2965-2774.20260105

SUPPLEMENTARY MATERIAL

Effects of pre-membrane acidification on extracorporeal CO_2 removal compared with conventional $ECCO_2R$ in mechanically ventilated animal models: a preclinical systematic review protocol

Vagner Pires de Campos Júnior[1], Anne Caroline Almeida de Sousa[1], Yuri de Albuquerque Pessoa dos Santos[2], Maristela Carvalho[3], Marcelo Amato Britto Passos[4], Eduardo Leite Vieira Costa[4]

Table 1S - Comprehensive search strategy for pre-membrane acidification and $ECCO_2R$

1.	MEDLINE®
#1	("Extracorporeal Circulation"[Mesh] OR "Extracorporeal Membrane Oxygenation"[Mesh] OR "Venovenous ECMO" OR Extracorporeal Life Support* OR "ECCO2R" OR "ECLS" OR "Extracorporeal carbon dioxide removal" OR "Venovenous extracorporeal membrane oxygenation")
#2	("Acidosis"[MESH] OR acids* OR "regional blood acidification" OR "pre-membrane acidification" OR "premembrane acidification" OR "lactic acid ")
#3	("carbon dioxide"[MESH] OR "VCO2" OR "CO2 removal" OR "CO2 elimination" OR "CO2 transfer" OR "carbon dioxide clearance" OR "carbon dioxide removal" OR "carbon dioxide elimination" OR "Carbon dioxide transfer" OR "Blood Gas Analysis"[MESH] OR Blood Gas Analys*)
#4	((Animals[Mesh] OR animal) AND ("Swine"[Mesh] OR "pig" OR "porcine" OR "Sheep"[Mesh] OR "lamb" OR "ovine" OR "Cattle"[Mesh] OR "cattle" OR "calf" OR "calves" OR "bovine" OR "cow" OR "Dogs"[Mesh] OR "dog" OR "canine" OR "Goats"[Mesh] OR "goat" OR caprine*)) OR ("Swine"[Mesh] OR "pig" OR "porcine" OR "Sheep"[Mesh] OR "lamb" OR "ovine" OR "Cattle"[Mesh] OR "cattle" OR "calf" OR "calves" OR "bovine" OR "cow" OR "Dogs"[Mesh] OR "dog" OR "canine" OR "Goats"[Mesh] OR "goat" OR caprine*)
#5	#1 AND #2 AND #3 AND #4
**2.**	**Scopus**
#1	"Extracorporeal Circulation" OR "Extracorporeal Membrane Oxygenation" OR "Venovenous ECMO" OR "Extracorporeal Life Support" OR "Extracorporeal Life Support*" OR "ECCO2R" OR "ECLS" OR "Extracorporeal carbon dioxide removal" OR "Venovenous extracorporeal membrane oxygenation"
#2	"Acidosis" OR "acid" OR regional blood acidification OR pre-membrane acidification OR premembrane acidification OR "lactic acid"
#3	"Animals" OR "Swine" OR "pig" OR "porcine" OR "Sheep" OR "lamb" OR "ovine" OR "Cattle" OR "cattle" OR "calf" OR "calves" OR "bovine" OR "cow" OR "Dogs" OR "dog" OR "canine" OR "Goats" OR "goat" OR "caprine*"
#4	TITLE-ABS-KEY ("Extracorporeal Circulation" OR "Extracorporeal Membrane Oxygenation" OR "Venovenous ECMO" OR "Extracorporeal Life Support" OR "Extracorporeal Life Support*" OR "ECCO2R" OR "ECLS" OR "Extracorporeal carbon dioxide removal" OR "Venovenous extracorporeal membrane oxygenation") AND TITLE-ABS-KEY ("Acidosis" OR "acid" OR regional blood acidification OR pre-membrane acidification OR premembrane acidification OR "lactic acid") AND TITLE-ABS-KEY ("Animals" OR "Swine" OR "pig" OR "porcine" OR "Sheep" OR "lamb" OR "ovine" OR "Cattle" OR "cattle" OR "calf" OR "calves" OR "bovine" OR "cow" OR "Dogs" OR "dog" OR "canine" OR "Goats" OR "goat" OR "caprine*")
**3.**	**Embase**
#1	'extracorporeal circulation'/exp OR 'extracorporeal oxygenation'/exp OR 'venovenous ecmo' OR 'extracorporeal life support*' OR 'ecco2r' OR 'ecls' OR 'extracorporeal carbon dioxide removal' OR 'venovenous extracorporeal membrane oxygenation'
#2	'acidosis'/exp OR 'acids*' OR 'regional blood acidification' OR 'pre-membrane acidification' OR 'premembrane acidification' OR 'lactic acid'
#3	'carbon dioxide'/exp OR 'vco2' OR 'co2 removal' OR 'co2 elimination' OR 'co2 transfer' OR 'carbon dioxide clearance' OR 'blood gas analysis'/exp OR 'blood gas analys*'
#4	'animal'/exp AND ('pig'/exp OR 'pig' OR 'porcine' OR 'sheep'/exp OR 'lamb' OR 'ovine' OR 'bovine'/exp OR 'cattle' OR 'calf' OR 'calves' OR 'bovine' OR 'cow' OR 'dog'/exp OR 'dog' OR 'canine' OR 'goat'/exp OR 'goat' OR 'caprine*'
#5	#1 AND #2 AND #3 AND #4

Continue...

This is an open access article under the CC BY license (https://creativecommons.org/licenses/by/4.0/).

...continuation

4.	Web of Science
#1	(Extracorporeal Circulation) OR (Extracorporeal Membrane Oxygenation) OR (Venovenous ECMO) OR (Extracorporeal Life Support*) OR ECCO2R OR ECLS OR (Extracorporeal carbon dioxide removal) OR (Venovenous extracorporeal membrane oxygenation)
#2	(Acidosis OR acids* OR (regional blood acidification) OR (pre-membrane acidification) OR (premembrane acidification) OR (lactic acid)))
#3	((carbon dioxide) OR VCO2 OR (CO2 removal) OR (CO2 elimination) OR (CO2 transfer) OR (carbon dioxide clearance) OR (Blood Gas Analysis) OR (Blood Gas Analysis))
#4	((Animals OR animal) AND ("Swine" OR "pig" OR "porcine" OR "Sheep" OR "lamb" OR "ovine" OR "Cattle" OR "cattle" OR "calf" OR "calves" OR "bovine" OR "cow" OR "Dogs" OR "dog" OR "canine" OR "Goats" OR "goat" OR caprine*))
#5	#1 AND #2 AND #3 AND #4
**5.**	**Google Scholar**
#1	((Extracorporeal Circulation) OR (Extracorporeal Membrane Oxygenation) OR (Venovenous ECMO) OR (Extracorporeal Life Support*) OR ECCO2R OR ECLS OR (Extracorporeal carbon dioxide removal) OR (Venovenous extracorporeal membrane oxygenation"))
#2	((Acidosis OR acids* OR (regional blood acidification) OR (pre-membrane acidification) OR (premembrane acidification) OR (lactic acid)))
#3	("carbon dioxide" OR "VCO2" OR "CO2 removal" OR "CO2 elimination" OR "CO2 transfer" OR "carbon dioxide clearance" OR "carbon dioxide removal" OR "carbon dioxide elimination" OR "Carbon dioxide transfer" OR "Blood Gas Analysis" OR Blood Gas Analys*)
#4	((Animals OR animal) AND ("Swine" OR "pig" OR "porcine" OR "Sheep" OR "lamb" OR "ovine" OR "Cattle" OR "cattle" OR "calf" OR "calves" OR "bovine" OR "cow" OR "Dogs" OR "dog" OR "canine" OR "Goats" OR "goat" OR caprine*))
#5	#1 AND #2 AND #3 AND #4
